# Effect of Intravitreal Brolucizumab in the Treatment of Polypoidal Choroidal Vasculopathy With Foveal Lipid Exudation

**DOI:** 10.7759/cureus.47942

**Published:** 2023-10-30

**Authors:** Valentina Carta, Filippo Lixi, Filippo Tatti, Enrico Peiretti

**Affiliations:** 1 Department of Surgical Sciences, Eye Clinic, University of Cagliari, Cagliari, ITA

**Keywords:** intravitreal, anti-vegf, brolucizumab, lipid exudation, polypoidal choroidal vasculopathy

## Abstract

A 71-year-old woman with a history of blurred vision in her right eye for nearly two months came to our attention. A complete ophthalmological evaluation, including best-corrected visual acuity measurement, fundus examination, spectral-domain optical coherence tomography, fluorescein angiography, and indocyanine green angiography, was performed. Multimodal imaging showed the presence of a polypoidal choroidal vasculopathy (PCV) lesion surrounded by diffuse hard exudates in the macular area. Our patient received three monthly intravitreal injections of brolucizumab during the loading phase, followed by an intravitreal injection every eight weeks for a total of 48 weeks of follow-up. The therapy appeared to be effective for improving both visual and anatomical outcomes revealing an important regression of the PCV and an almost complete reabsorption of lipid exudates. Intravitreal brolucizumab could be considered an effective treatment in the management of lipid exudation in PCV patients.

## Introduction

Polypoidal choroidal vasculopathy (PCV) is a choroidal neovascular disorder, first described by Yannuzzi et al. in the 1990s. Originally, PCV was considered a subtype of exudative age-related macular degeneration (AMD) with similar clinical and genetic features [1]. Furthermore, the presence of thick choroids has led to include PCV in the pachychoroid spectrum, as well as it has been described as associated with many primary chorioretinal diseases, such as tilted disc syndrome, choroidal nevus, and sickle cell retinopathy [2-4].

PCV is considered to be more common in Asian populations, and recent studies have shown an increasing prevalence in Caucasian patients [5]. Clinically, PCV is characterized by visual loss secondary to a serosanguinous maculopathy, where lipid exudation, subretinal blood, and the presence of orange nodules represent the main features on fundus examination. Moreover, on multimodal imaging, the hallmark of the disease is a branching neovascular network (BNN) with abnormal aneurysmal dilatations referred to as polypoidal lesions [5,6].

Although several techniques such as fluorescein angiography (FAG), optical coherence tomography (OCT), and optical coherence tomography angiography (OCTA) are commonly used, indocyanine green angiography (ICGA) is still considered the gold standard for the diagnosis of PCV [6].

Different therapeutic options such as laser treatment, photodynamic therapy (PDT), and anti-vascular endothelial growth factor (anti-VEGF) injections alone or in combination have been proposed [5,6]. Among these, anti-VEGF agents have been demonstrated highly effective for PCV regression and fluid control [7]. Furthermore, anti-VEGF with PDT showed some effect in lipid exudation in PCV eyes, while in this report, the presence of subfoveal exudates was considered a predictive factor for poor outcomes at one year [8].

The recently developed anti-VEGF, brolucizumab (Beovu; Novartis, London, UK), a humanized, single-chain variable fragment that inhibits all isoforms of vascular endothelial growth factor A (VEGF A), showed best-corrected visual acuity (BCVA) gains, superior anatomical outcomes, and a lower treatment burden compared to other anti-VEGF agents in PCV patients [9-11].

Herein, we describe a case of PCV exhibiting a dramatic clinical response to brolucizumab injections, especially considering the lipid exudation reabsorption.

## Case presentation

**Figure 1:**
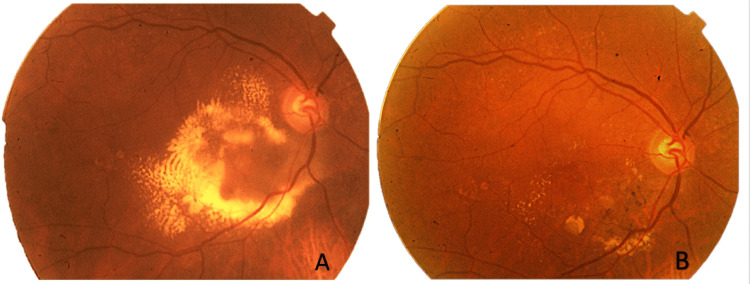
Fundus photo of the right eye showing lipid exudation changes before treatment (A) and at week 48 (B). The images demonstrate dense intraretinal hard exudates concentrated in the macular area and inferonasally (A), which drastically reduced after treatment (B).

A 71-year-old Caucasian woman was referred to the retina medical unit for a progressive and painless visual loss in her right eye (RE) for two months duration. The patient did not report any previous ocular surgery, trauma, or eye inflammation history. A slit lamp examination showed moderate subcapsular sclerotic changes in the right lens and initial lens sclerosis in the left eye (LE). The intraocular pressure was 18 mmHg in both eyes and the BCVA was 20/400 in the RE and 20/20 in the LE. Funduscopic examination of the RE revealed yellowish lipid exudates diffuse in the macular and foveal area (Figure 1).

**Figure 2:**
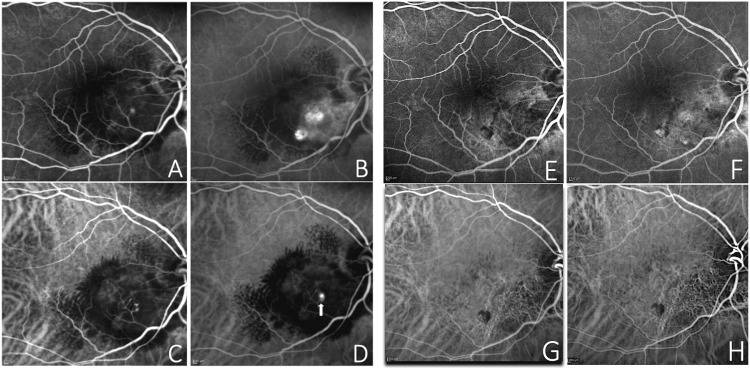
Fundus fluorescein angiography (FA) at baseline (A-B) showing some leakage (A) and granular areas of hyperfluorescence increasing at late times inferonasally to the fovea (B). Indocyanine green angiography (ICGA) at baseline (C-D) displaying some dot-like hypercianescence visible in the early phase (C), which increased in the late phase (D) corresponding to the polyp (white arrow). FA (E-F) and ICGA (G-H) at week 48 showing a complete regression of the polypoidal lesion.

LE fundus examination was unremarkable. FAG (Heidelberg Engineering, Heidelberg, Germany) of the RE showed granular hyperfluorescence increasing in late times inferonasally to the fovea and diffuse hypofluorescence in the macular area due to lipid exudation. ICGA (Heidelberg Engineering) confirmed the presence of a polypoidal choroidal neovascularization showing a dot-like hyperfluorescent spot increasing in late times (Figure 2).

**Figure 3:**
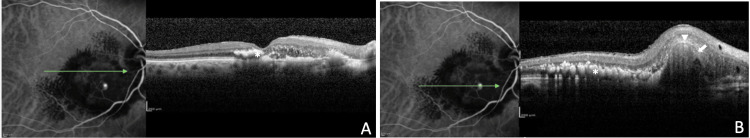
Spectral-domain OCT scans at baseline, obtained through the fovea (A), imaged in ICGA revealing multiple intraretinal hard exudates (*). The ICGA-guided OCT scan (B) shows the presence of an intraretinal hyporeflective space, within the sharp pigment epithelial detachment (PED) (arrowhead), which contains a hyporeflective lumen nasally (white arrow). OCT: optical coherence tomography; ICGA: indocyanine green angiography.

Spectral-domain optical coherence tomography (SD-OCT, Heidelberg Engineering) of the RE evidenced diffuse intraretinal hyperreflective lesions, such as an important lipid deposition in the macular area, intraretinal hyporeflective cystoid spaces representing intraretinal fluid (IRF), and a sharp pigment epithelial detachment (PED) peak (thumb-like) corresponding to the polyp inferonasally to the fovea (Figure 3).

**Figure 4:**
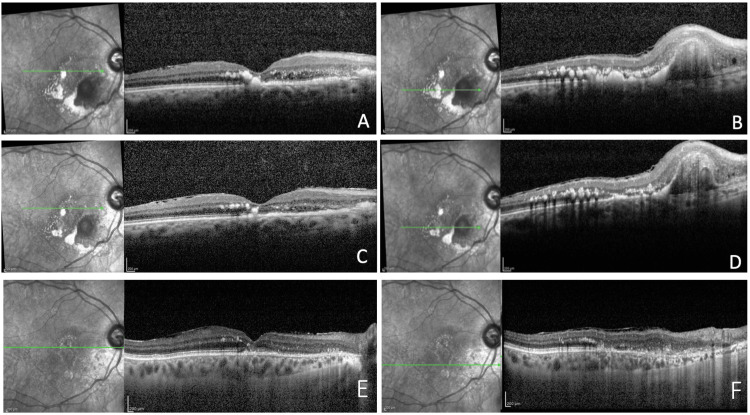
Spectral-domain optical coherence tomography scans demonstrate the evolution of the lesion during the follow-up period. After the loading dose, images show a regression of intraretinal hard exudates, a reduction of intraretinal hyporeflective cystoid spaces and of the polypoidal lesion (A-B). At six months follow-up, intraretinal hard exudates are considerably reduced in number and size, some intraretinal hyporeflective cystoid spaces are still present and the polypoidal lesion appeared reduced in height (C-D). At 48 weeks, images highlight the drastic reduction of lipid exudation throughout the macular area. The complete disappearance of foveal hard exudate with the restoration of the integrity of the ELM (external limiting membrane)-EZ (ellipsoid zone) complex looks dramatic and a complete resolution of intraretinal fluid cysts and a significative regression in height of the polypoidal lesion (E-F) was also obtained.

The patient underwent a loading dose of intravitreal brolucizumab 6.0 mg/0.05 mL (one every four weeks for the first three months) followed by q8 weekly retreatment regimen for 48 weeks (every eight weeks of follow-up) for a total of eight intravitreal injections. A complete ophthalmologic examination was performed one week and one month after each injection to rule out possible intraocular inflammations (IOI). After the loading dose, a fundus exam of the RE showed an initial reduction of hard exudates in the macular area, confirmed by an OCT scan, which also displayed a lowering of intraretinal hyporeflective cysts and a partial shrinking of the PED corresponding to the polypoidal lesion (Figures 4A, 4B).

At the six-month follow-up, the patient had a visual acuity of 20/80, fundus examination of the RE evidenced a significative reabsorption of lipid exudates in the macular area, and OCT showed a drastic decrease of hyperreflective lesions, accompanied by an important reabsorption of IRF and a lesion’s height lowering (Figures 4C, 4D).

At the 48-week follow-up visit, a fundus examination of the RE evidenced an almost complete reduction of lipid exudation at the posterior pole (Figure 1B). OCT demonstrated a resolution of IRF (Figures 4E, 4F). FAG and ICGA examinations uncovered a complete regression of the polypoidal lesion (Figures 2E-2H). Despite the subcapsular sclerotic changes in the lens, the patient had a visual acuity improvement to 20/60 in the RE. No ocular or systemic adverse events related to the use of the drug were observed.

## Discussion

Hard exudates are deposits, localized generally in the outer plexiform layer of the retina, composed of lipid and proteinaceous material, such as fibrinogen and albumin, which leak from impaired vessels as a result of the breakdown of the blood-retina barrier [12]. Since severe vision loss may occur when lipid exudates are localized at the macula, understanding its pathogenesis and evaluating the most suitable treatment represent an urgent need [13].

In PCV patients, retinal pigment epithelium (RPE) decompensation and persistent neurosensory retinal detachment can determine lipid exudation from neovascular tissue [13].

Currently, vascular endothelial growth factor (VEGF), as a mediator of choroidal neovascularization and vascular permeability, is considered implicated in the development of PCV. Some studies reported VEGF expression in vascular endothelial and RPE cells analyzing surgical specimens and higher VEGF levels in aqueous humor in eyes with PCV compared with controls, although much lower than in eyes with typical neovascular AMD (nAMD) [14,15].

Anti-VEGF drugs have been demonstrated to reduce leakage from abnormal polyps and choroidal vessels, improving macular edema and visual acuity [7]. Furthermore, in some studies, anti-VEGF agents have proved to be effective in reducing hard exudates in retinal diseases such as diabetic retinopathy, retinal vein occlusion, nAMD, and Coats disease [16-18].

Besides the regression of the polypoidal lesion and the resolution of IRF, our case showed massive and rapid reabsorption of lipid exudates after brolucizumab injections. According to the literature, the HAWK PCV subpopulation analysis showed a greater reduction of IRF in brolucizumab-treated eyes compared with aflibercept in Japanese patients [14].

A recent study by Ito et al. confirmed the efficacy of brolucizumab evidencing a higher regression of polypoidal lesions in comparison with ranibizumab and aflibercept [19]. Therefore, although we could have expected a good anatomical response, the presence of lipid exudation at baseline in the fovea represented a negative prognostic factor [8]. The exudates’ reabsorption in the macular area in only six months allowed a significant final visual gain improvement in our patient. Nonetheless, the mechanism by which brolucizumab effectively and rapidly reduces hard exudates remains speculative.

The smaller size of brolucizumab, compared to other anti-VEFG agents, enables more drug delivery per injection and better tissue penetration in the neurosensory retina and retinal pigment epithelium [9]. This could partially explain the strong control on vascular permeability and consequentially the reduction of material leakage from choroidal vessels. Moreover, brolucizumab has been demonstrated to dry the macula faster and better in patients with nAMD than aflibercept [20]. Consequently, the action on the neovascular membrane allows not only the resolution of the nonlipid fluid components but also considerable and rapid reabsorption of the lipid exudates.

To our knowledge, this is the first report that highlights the effect of brolucizumab monotherapy in reducing lipid exudation in the macular area of a PCV lesion.

## Conclusions

In conclusion, intravitreal brolucizumab can be considered a valuable treatment option in patients with PCV with an important component of intraretinal hard exudates. Nevertheless, prospective studies with longer follow-up and a bigger cohort are needed to confirm the efficacy and safety of this relatively novel treatment strategy.
